# Supplementary material for: Inactivation of Bacteria and Residual Antimicrobials in Hospital Wastewater by Ozone Treatment
Source: Antibiotics (Basel). 2022 Jun 27;11(7):862. doi: 10.3390/antibiotics11070862 (PMC9311624; doi:10.3390/antibiotics11070862)
Supplement: Supplementary file 1 [file antibiotics-11-00862-s001.zip › Supplementary-Table-S1.pdf]

antibiotics

MDPI

Supplementary Materials:

Table S1. Summary of water quality parameters during hospital wastewater treatment with ozone (N.D.: Not detected).

Water quality items	Treatment time (min)		
	0	180	360
COD (mg/L)	228	238	205
BOD (mg/L)	359	372	355
SS (mg/L)	483	402	411
Coliform group (CFU/mL)	25000	11	2
Injected gas ozone concentration (mg/L)	103	103	103
Waste gas ozone concentration (mg/L)	N.D.	N.D.	4011
Dissolved ozone concentration (mg/L)	N.D.	0.06	0.09
