# Supplementary material for: Inactivation of Bacteria and Residual Antimicrobials in Hospital Wastewater by Ozone Treatment
Source: Antibiotics (Basel). 2022 Jun 27;11(7):862. doi: 10.3390/antibiotics11070862 (PMC9311624; doi:10.3390/antibiotics11070862)
Supplement: Supplementary file 1 [file antibiotics-11-00862-s001.zip › Supplementary-Table-S2.pdf]

antibiotics

MDPI

Supplementary Materials:

Table S2. Validation of the method characteristics for analysis of antimicrobials in hospital wastewater.

Classification	Antimicrobials	Recovery (%) (SD, n=3)	LOD (ng/L)	LOQ (ng/L)	Calibration range (ng/mL)	Correlation coefficient (r^2)
β-lactams	Ampicillin	48 (6)	1.6	5.4	0.5–200	0.99
	Cefdinir	87 (7)	0.2	0.8	0.5–200	0.99
	Cefpodoxime	91 (5)	0.3	1.1	0.5–200	0.99
	Cefpodoxime proxetil	91 (6)	1.2	3.9	0.5–200	0.99
	Ceftiofur	96 (7)	0.6	2.1	0.5–200	0.99
New quinolones	Ciprofloxacin	86 (6)	0.7	2.2	0.5–200	0.99
	Levofloxacin	83 (9)	0.3	1.0	0.5–200	0.99
Macrolides	Azithromycin	97 (7)	0.3	1.1	0.5–200	0.99
	Clarithromycin	53 (10)	0.5	1.6	0.5–200	0.99
Tetracyclines	Chlortetracycline	90 (5)	0.3	1.1	0.5–200	0.99
	Doxycycline	96 (3)	0.3	1.0	0.5–200	0.99
	Minocycline	98 (4)	0.3	1.0	0.5–200	0.99
	Oxytetracycline	85 (6)	0.3	1.1	0.5–200	0.99
	Tetracycline	65 (6)	0.4	1.3	0.5–200	0.99
Glycopeptide	Vancomycin	92 (9)	0.6	1.9	0.5–200	0.99
