# Supplementary material for: Inactivation of Bacteria and Residual Antimicrobials in Hospital Wastewater by Ozone Treatment
Source: Antibiotics (Basel). 2022 Jun 27;11(7):862. doi: 10.3390/antibiotics11070862 (PMC9311624; doi:10.3390/antibiotics11070862)
Supplement: Supplementary file 1 [file antibiotics-11-00862-s001.zip › Supplementary-Table-S3.pdf]

antibiotics

Supplementary Materials:

Table S3. Comparison of RPKM value after ozone treatment.

		RPKM value for each OTU of ARG (AMROTU) after ozone treatment				
**AMROTU_ID**	**ARG list**	**0 min**	**10 min**	**20 min**	**40 min**	**80 min**
AMROTU_38	tet(Q)	58.5	73.2	0.0	0.0	0.0
AMROTU_550	blaGES-1, blaGES-10, blaGES-11, blaGES-12, blaGES-13, blaGES-14, blaGES-15, blaGES-16, blaGES-17, blaGES-18, blaGES-19, blaGES-2, blaGES-20, blaGES-21, blaGES-22, blaGES-24, blaGES-25, blaGES-26, blaGES-27, blaGES-28, blaGES-29, blaGES-3, blaGES-30, blaGES-31, blaGES-32, blaGES-33, blaGES-34, blaGES-35, blaGES-36, blaGES-37, blaGES-38, blaGES-39, blaGES-4, blaGES-40, blaGES-41, blaGES-42, blaGES-43, blaGES-44, blaGES-45, blaGES-46, blaGES-47, blaGES-48, blaGES-49, blaGES-5, blaGES-50, blaGES-51, blaGES-6, blaGES-7, blaGES-8, blaGES-9	57.3	75.0	0.0	0.0	0.0
AMROTU_47	tet(O/W), tet(O/W)-1, tet(W), tet(W/N/W)	26.2	25.0	0.0	0.0	0.0
AMROTU_674	aph(3'')-Ib	24.7	40.8	0.0	0.0	0.0
AMROTU_1252	qacF, qacL	24.4	7.8	0.0	0.0	0.0
AMROTU_1012	aac(6')-Ib, aac(6')-Ib', aac(6')-Ib11, aac(6')-Ib3, aac(6')-Ib4, aac(6')-Ib-cr, aac(6')Ib-cr, aac(6')-Ib-cr10, aac(6')-Ib-cr11, aac(6')-Ib-cr3, aac(6')-Ib-cr4, aac(6')-Ib-cr5, aac(6')-Ib-cr6, aac(6')-Ib-cr7, aac(6')-Ib-cr8, aac(6')-Ib-cr9, aac(6')-Ib-Hangzhou	23.9	25.3	0.0	0.0	0.0
AMROTU_360	aadA1, aadA11, aadA12, aadA15, aadA1bt, aadA21, aadA22, aadA23, aadA24, aadA25, ant(3'')-Ia	18.6	10.3	0.0	0.0	0.0
AMROTU_525	sul1	18.4	30.8	0.0	0.0	0.0
AMROTU_658	aph(6)-Id	14.1	44.7	0.0	0.0	0.0
AMROTU_485	cblA	14.1	8.3	0.0	0.0	0.0
AMROTU_1226	fosE	13.6	1.9	0.0	0.0	0.0
AMROTU_1251	qacE	11.1	21.1	0.0	0.0	0.0
AMROTU_383	qacH	11.0	11.8	0.0	0.0	0.0
AMROTU_50	tet(O), tet(O/32/O), tet(O/M/O)	10.8	7.5	0.0	0.0	0.0

AMROTU_742	blaOXA-10, blaOXA-101, blaOXA-1015, blaOXA-1046, blaOXA-1053, blaOXA-1086, blaOXA-11, blaOXA-13, blaOXA-14, blaOXA-142, blaOXA-145, blaOXA-147, blaOXA-16, blaOXA-17, blaOXA-183, blaOXA-19, blaOXA-233, blaOXA-240, blaOXA-246, blaOXA-251, blaOXA-256, blaOXA-28, blaOXA-35, blaOXA-368, blaOXA-454, blaOXA-520, blaOXA-56, blaOXA-655, blaOXA-656, blaOXA-663, blaOXA-676, blaOXA-677, blaOXA-7, blaOXA-736, blaOXA-74, blaOXA-794, blaOXA-795, blaOXA-823, blaOXA-824, blaOXA-827, blaOXA-836, blaOXA-928, blaOXA-932, blaOXA-935	10.2	2.3	0.0	0.0	0.0
AMROTU_1046	aac(6')-31, aacA8	9.4	15.6	0.0	0.0	0.0
AMROTU_844	erm(B)	9.0	9.5	0.0	0.0	0.0
AMROTU_117	msr(E)	7.0	9.0	0.0	0.0	0.0
AMROTU_1088	ant(2'')-Ia	6.9	5.5	0.0	0.0	0.0
AMROTU_52	tet(O/W/32/O), tet(W/32/O)	6.5	8.3	0.0	0.0	0.0
AMROTU_711	aadA11, aadA12, aadA17, aadA2, aadA25, aadA2b, aadA3, aadA8	6.5	5.1	0.0	0.0	0.0
AMROTU_277	blaEC-13, blaEC-14, blaEC-15, blaEC-16, blaEC-18, blaEC-19, blaEC-5, blaEC-8	5.9	0.7	0.0	0.0	0.0
AMROTU_1101	lnu(AN2)	5.8	11.6	0.0	0.0	0.0
AMROTU_764	erm(F)	5.5	17.6	0.0	0.0	0.0
AMROTU_371	cfxA, cfxA2, cfxA3, cfxA4, cfxA5	5.4	13.0	0.0	0.0	0.0
AMROTU_197	tet(39)	5.1	6.1	0.0	0.0	0.0
AMROTU_49	tet(32)	5.0	2.5	0.0	0.0	0.0
AMROTU_873	blaIMP-1, blaIMP-10, blaIMP-25, blaIMP-26, blaIMP-3, blaIMP-30, blaIMP-34, blaIMP-38, blaIMP-4, blaIMP-40, blaIMP-42, blaIMP-43, blaIMP-51, blaIMP-52, blaIMP-55, blaIMP-59, blaIMP-6, blaIMP-60, blaIMP-61, blaIMP-66, blaIMP-7, blaIMP-70, blaIMP-73, blaIMP-76, blaIMP-77, blaIMP-78, blaIMP-79, blaIMP-80, blaIMP-88, blaIMP-89, blaIMP-94	5.0	2.5	0.0	0.0	0.0
AMROTU_172	tet(40)	4.8	8.2	0.0	0.0	0.0
AMROTU_670	blaOXA-1017, blaOXA-1049, blaOXA-1082, blaOXA-1083, blaOXA-1084, blaOXA-1085, blaOXA-141, blaOXA-15, blaOXA-161, blaOXA-2, blaOXA-21, blaOXA-210, blaOXA-226, blaOXA-3, blaOXA-32, blaOXA-34, blaOXA-36, blaOXA-415, blaOXA-539, blaOXA-540, blaOXA-541, blaOXA-543, blaOXA-544, blaOXA-681, blaOXA-737, blaOXA-838	4.5	3.2	0.0	0.0	0.0
AMROTU_1119	dfrF	4.5	2.2	0.0	0.0	0.0
AMROTU_1253	qacG2	4.4	1.1	0.0	0.0	0.0

AMROTU_579	aadS	4.3	1.3	0.0	0.0	0.0
AMROTU_570	aadE	4.3	7.7	0.0	0.0	0.0
AMROTU_180	mef(En2)	3.7	7.1	0.0	0.0	0.0
AMROTU_744	vanY-B	3.7	0.9	0.0	0.0	0.0
AMROTU_178	floR	3.7	0.6	0.0	0.0	0.0
AMROTU_714	aph(3')-Ia	3.6	0.0	0.0	0.0	0.0
AMROTU_167	mef(B)	3.6	3.0	0.0	0.0	0.0
AMROTU_615	erm(X)	3.6	2.2	0.0	0.0	0.0
AMROTU_999	catA13	3.6	1.8	0.0	0.0	0.0
AMROTU_544	blaORN-1, blaORN-2, blaORN-3, blaORN-4, blaORN-5, blaORN-6, blaPLA-1, blaPLA-2a, blaPLA-3, blaPLA-4, blaPLA-5, blaPLA-6	3.4	1.7	0.0	0.0	0.0
AMROTU_514	mph(E)	3.3	2.9	0.0	0.0	0.0
AM-ROTU_1174	aac(6')-Il	3.2	4.0	0.0	0.0	0.0
AMROTU_131	ant(3'')-Ih/aac(6')-IId, ant(3'')-Ij/aac(6')-Ib	3.2	1.3	0.0	0.0	0.0
AMROTU_810	blaLCR-1, blaNPS-1	2.8	4.7	0.0	0.0	0.0
AMROTU_240	blaMOX-9	2.6	0.0	0.0	0.0	0.0
AMROTU_195	tet(C)	2.5	2.5	0.0	0.0	0.0
AMROTU_146	tet(A)	2.5	1.8	0.0	0.0	0.0
AM-ROTU_1005	vanX-B	2.4	1.2	0.0	0.0	0.0
AMROTU_44	tet(M), tet(S/M)	2.3	3.1	0.0	0.0	0.0
AMROTU_346	cfxA6	2.3	0.8	0.0	0.0	0.0
AMROTU_943	vanR-B	2.2	1.1	0.0	0.0	0.0
AM-ROTU_1247	qacEdelta1	2.1	13.8	0.0	0.0	0.0
AMROTU_893	erm(G)	2.0	5.5	0.0	0.0	0.0

AMROTU_888	blaTMB-1, blaTMB-2	2.0	1.0	0.0	0.0	0.0
AMROTU_115	msr(C)	2.0	0.0	0.0	0.0	0.0
AMROTU_171	tet(33)	1.8	1.8	0.0	0.0	0.0
AMROTU_664	blaOXA-299	1.8	0.0	0.0	0.0	0.0
AMROTU_609	blaACI-1	1.7	0.9	0.0	0.0	0.0
AMROTU_584	blaTEM-1, blaTEM-10, blaTEM-101, blaTEM-102, blaTEM-103, blaTEM-104, blaTEM-105, blaTEM-106, blaTEM-107, blaTEM-108, blaTEM-109, blaTEM-11, blaTEM-110, blaTEM-111, blaTEM-112, blaTEM-113, blaTEM-114, blaTEM-115, blaTEM-116, blaTEM-12, blaTEM-120, blaTEM-121, blaTEM-122, blaTEM-123, blaTEM-124, blaTEM-125, blaTEM-126, blaTEM-127, blaTEM-128, blaTEM-129, blaTEM-130, blaTEM-131, blaTEM-132, blaTEM-133, blaTEM-134, blaTEM-135, blaTEM-136, blaTEM-137, blaTEM-138, blaTEM-139, blaTEM-141, blaTEM-142, blaTEM-143, blaTEM-144, blaTEM-145, blaTEM-146, blaTEM-147, blaTEM-148, blaTEM-149, blaTEM-15, blaTEM-150, blaTEM-151, blaTEM-152, blaTEM-153, blaTEM-154, blaTEM-155, blaTEM-156, blaTEM-157, blaTEM-158, blaTEM-159, blaTEM-16, blaTEM-160, blaTEM-162, blaTEM-163, blaTEM-164, blaTEM-166, blaTEM-167, blaTEM-168, blaTEM-169, blaTEM-17, blaTEM-171, blaTEM-176, blaTEM-177, blaTEM-178, blaTEM-181, blaTEM-182, blaTEM-183, blaTEM-184, blaTEM-185, blaTEM-186, blaTEM-187, blaTEM-188, blaTEM-189, blaTEM-19, blaTEM-190, blaTEM-191, blaTEM-193, blaTEM-194, blaTEM-195, blaTEM-196, blaTEM-197, blaTEM-198, blaTEM-1A, blaTEM-1C, blaTEM-1D, blaTEM-2, blaTEM-20, blaTEM-201, blaTEM-205, blaTEM-206, blaTEM-207, blaTEM-208, blaTEM-209, blaTEM-21, blaTEM-210, blaTEM-211, blaTEM-212, blaTEM-213, blaTEM-214, blaTEM-215, blaTEM-216, blaTEM-217, blaTEM-219, blaTEM-22, blaTEM-220, blaTEM-224, blaTEM-225, blaTEM-226, blaTEM-227, blaTEM-228, blaTEM-229, blaTEM-230, blaTEM-231, blaTEM-232, blaTEM-233, blaTEM-234, blaTEM-235, blaTEM-236, blaTEM-237, blaTEM-238, blaTEM-239, blaTEM-24, blaTEM-240, blaTEM-241, blaTEM-242, blaTEM-243, blaTEM-244, blaTEM-245, blaTEM-246, blaTEM-26, blaTEM-28, blaTEM-29, blaTEM-3, blaTEM-30, blaTEM-31, blaTEM-32, blaTEM-33, blaTEM-34, blaTEM-35, blaTEM-36, blaTEM-37, blaTEM-39, blaTEM-4, blaTEM-40, blaTEM-43, blaTEM-45, blaTEM-47, blaTEM-48, blaTEM-49, blaTEM-5, blaTEM-52, blaTEM-52B, blaTEM-52C, blaTEM-53, blaTEM-54, blaTEM-55, blaTEM-57, blaTEM-6, blaTEM-60, blaTEM-61, blaTEM-63, blaTEM-67, blaTEM-68, blaTEM-7, blaTEM-70, blaTEM-71, blaTEM-72, blaTEM-76, blaTEM-77, blaTEM-78, blaTEM-79, blaTEM-8, blaTEM-80, blaTEM-81, blaTEM-82, blaTEM-83, blaTEM-84, blaTEM-85, blaTEM-86, blaTEM-87, blaTEM-88, blaTEM-9, blaTEM-90, blaTEM-91, blaTEM-92, blaTEM-93, blaTEM-94, blaTEM-95, blaTEM-96, blaTEM-97, blaTEM-98, blaTEM-99	1.7	0.0	0.0	0.0	0.0
AMROTU_454	mph(A)	1.6	4.9	0.0	0.0	0.0
AMROTU_119	msr(D)	1.5	0.8	0.0	0.0	0.0
AMROTU_4	sitABCD	1.5	2.1	0.0	0.0	0.0
AMROTU_275	blaSRT, blaSRT-1, blaSRT-2, blaSRT-3, blaSST-1	1.3	0.0	0.0	0.0	0.0
AMROTU_251	ampC	1.3	0.0	0.0	0.0	0.0
AMROTU_218	tet(X2), tet(X4)	1.3	2.9	0.0	0.0	0.0
AMROTU_175	tet(E)	1.2	0.0	0.0	0.0	0.0
AMROTU_169	mef(A)	1.2	1.8	0.0	0.0	0.0

AMROTU_154	ere(A), ere(A2)	1.2	0.0	0.0	0.0	0.0
AMROTU_5	oqxB, oqxB10, oqxB11, oqxB12, oqxB13, oqxB14, oqxB15, oqxB16, oqxB17, oqxB18, oqxB19, oqxB2, oqxB20, oqxB21, oqxB22, oqxB23, oqxB24, oqxB25, oqxB26, oqxB27, oqxB28, oqxB29, oqxB3, oqxB30, oqxB31, oqxB32, oqxB4, oqxB5, oqxB6, oqxB7, oqxB8, oqxB9	1.2	0.5	0.0	0.0	0.0
AMROTU_140	vanS-B, vanS-B-Delta	1.1	0.0	0.0	0.0	0.0
AMROTU_863	cfiA, cfiA10, cfiA11, cfiA13, cfiA14, cfiA17, cfiA18, cfiA19, cfiA2, cfiA21, cfiA22, cfiA23, cfiA24, cfiA26, cfiA27, cfiA28, cfiA3, cfiA4, cfiA6, cfiA8, cfiA9	1.0	5.4	0.0	0.0	0.0
AMROTU_794	aadA4, aadA5	0.9	4.7	0.0	0.0	0.0
AMROTU_779	blaOXA-1037, blaOXA-504, blaOXA-780	0.9	4.2	0.0	0.0	0.0
AMROTU_735	blaVIM-1, blaVIM-10, blaVIM-11, blaVIM-12, blaVIM-14, blaVIM-15, blaVIM-16, blaVIM-17, blaVIM-18, blaVIM-19, blaVIM-2, blaVIM-20, blaVIM-23, blaVIM-24, blaVIM-25, blaVIM-26, blaVIM-27, blaVIM-28, blaVIM-29, blaVIM-3, blaVIM-30, blaVIM-31, blaVIM-32, blaVIM-33, blaVIM-34, blaVIM-35, blaVIM-36, blaVIM-37, blaVIM-38, blaVIM-39, blaVIM-4, blaVIM-40, blaVIM-41, blaVIM-42, blaVIM-43, blaVIM-44, blaVIM-45, blaVIM-46, blaVIM-48, blaVIM-50, blaVIM-51, blaVIM-52, blaVIM-53, blaVIM-54, blaVIM-55, blaVIM-56, blaVIM-57, blaVIM-58, blaVIM-59, blaVIM-6, blaVIM-60, blaVIM-62, blaVIM-63, blaVIM-64, blaVIM-65, blaVIM-66, blaVIM-67, blaVIM-68, blaVIM-70, blaVIM-72, blaVIM-73, blaVIM-74, blaVIM-75, blaVIM-76, blaVIM-77, blaVIM-78, blaVIM-79, blaVIM-8, blaVIM-80, blaVIM-9	0.9	3.2	0.0	0.0	0.0
AMROTU_759	blaOXA-20, blaOXA-37	0.9	0.0	0.0	0.0	0.0
AMROTU_697	blaOXA-347	0.9	1.8	0.0	0.0	0.0
AMROTU_667	blaOXA-1, blaOXA-1042, blaOXA-224, blaOXA-31, blaOXA-320, blaOXA-392, blaOXA-4, blaOXA-47, blaOXA-534, blaOXA-675, blaOXA-796, blaOXA-921	0.9	4.5	0.0	0.0	0.0
AMROTU_64	tetA(46)	0.9	0.4	0.0	0.0	0.0
AMROTU_545	blaOXY-1-1, blaOXY-1-2, blaOXY-1-3, blaOXY-1-4, blaOXY-1-5, blaOXY-1-6, blaOXY-4-1, blaOXY-5-1, blaOXY-5-2, blaOXY-6-1, blaOXY-6-2, blaOXY-6-3, blaOXY-6-4	0.8	0.0	0.0	0.0	0.0
AMROTU_531	blaCTX-M-102, blaCTX-M-104, blaCTX-M-105, blaCTX-M-110, blaCTX-M-111, blaCTX-M-112, blaCTX-M-113, blaCTX-M-121, blaCTX-M-122, blaCTX-M-125, blaCTX-M-126, blaCTX-M-129, blaCTX-M-13, blaCTX-M-130, blaCTX-M-134, blaCTX-M-137, blaCTX-M-14, blaCTX-M-140, blaCTX-M-147, blaCTX-M-148, blaCTX-M-14b, blaCTX-M-159, blaCTX-M-16, blaCTX-M-161, blaCTX-M-168, blaCTX-M-17, blaCTX-M-174, blaCTX-M-19, blaCTX-M-191, blaCTX-M-192, blaCTX-M-195, blaCTX-M-196, blaCTX-M-198, blaCTX-M-201, blaCTX-M-21, blaCTX-M-213, blaCTX-M-214, blaCTX-M-215, blaCTX-M-219, blaCTX-M-221, blaCTX-M-223, blaCTX-M-233, blaCTX-M-235, blaCTX-M-239, blaCTX-M-24, blaCTX-M-240, blaCTX-M-241, blaCTX-M-242, blaCTX-M-243, blaCTX-M-252, blaCTX-M-27, blaCTX-M-38, blaCTX-M-46, blaCTX-M-47, blaCTX-M-48, blaCTX-M-49, blaCTX-M-50, blaCTX-M-51, blaCTX-M-65, blaCTX-M-67, blaCTX-M-73, blaCTX-M-81, blaCTX-M-83, blaCTX-M-84, blaCTX-M-85, blaCTX-M-86, blaCTX-M-87, blaCTX-M-9, blaCTX-M-90, blaCTX-M-93, blaCTX-M-98, blaCTX-M-99	0.8	1.3	0.0	0.0	0.0
AMROTU_517	bla	0.8	1.7	0.0	0.0	0.0
AMROTU_439	ant(6)-Ia	0.8	0.4	0.0	0.0	0.0

AMROTU_53	tet(O/W/O), tet(O/W/O)-1	0.8	0.6	0.0	0.0	0.0
AMROTU_51	tet(O/32/O)	0.8	0.4	0.0	0.0	0.0
AMROTU_244	blaCMY-1, blaCMY-10, blaCMY-11, blaCMY-19, blaCMY-8, blaCMY-8b, blaCMY-9, blaMOX-1, blaMOX-10, blaMOX-11, blaMOX-14, blaMOX-16, blaMOX-2, blaMOX-3, blaMOX-4	0.6	0.6	0.0	0.0	0.0
AMROTU_151	catB8/aac(6')-Ib'	0.6	0.9	0.0	0.0	0.0
AMROTU_63	tetB(46)	0.4	0.0	0.0	0.0	0.0
AMROTU_45	tet(36)	0.4	0.2	0.0	0.0	0.0
AMROTU_775	aph(3')-IIIa	0.0	5.1	0.0	0.0	0.0
AMROTU_818	aad9	0.0	3.8	0.0	0.0	0.0
AMROTU_622	sul2	0.0	3.6	0.0	0.0	0.0
AM-ROTU_1080	sat4	0.0	3.4	0.0	0.0	0.0
AM-ROTU_1153	dfrA1	0.0	3.1	0.0	0.0	0.0
AMROTU_588	aac(3)-IIa, aac(3)-IIc, aac(3)-IId, aac(3)-IIe	0.0	2.6	0.0	0.0	0.0
AM-ROTU_1023	aac(6')-IIc	0.0	2.5	0.0	0.0	0.0
AMROTU_202	tet(G)	0.0	2.5	0.0	0.0	0.0
AMROTU_173	mef(A)	0.0	2.4	0.0	0.0	0.0
AMROTU_756	blaMYO-1	0.0	2.3	0.0	0.0	0.0
AMROTU_957	qnrVC4, qnrVC5, qnrVC7, qnrVC9	0.0	1.7	0.0	0.0	0.0
AM-ROTU_1136	dfrA14	0.0	1.6	0.0	0.0	0.0
AM-ROTU_1124	catS	0.0	1.5	0.0	0.0	0.0
AMROTU_716	aph(3')-Ib	0.0	1.4	0.0	0.0	0.0

AMROTU_507	blaCTX-M-1, blaCTX-M-10, blaCTX-M-101, blaCTX-M-103, blaCTX-M-114, blaCTX-M-116, blaCTX-M-117, blaCTX-M-12, blaCTX-M-127, blaCTX-M-136, blaCTX-M-138, blaCTX-M-139, blaCTX-M-142, blaCTX-M-143, blaCTX-M-144, blaCTX-M-146, blaCTX-M-15, blaCTX-M-150, blaCTX-M-154, blaCTX-M-155, blaCTX-M-156, blaCTX-M-157, blaCTX-M-158, blaCTX-M-162, blaCTX-M-163, blaCTX-M-164, blaCTX-M-166, blaCTX-M-167, blaCTX-M-169, blaCTX-M-170, blaCTX-M-172, blaCTX-M-173, blaCTX-M-175, blaCTX-M-176, blaCTX-M-177, blaCTX-M-178, blaCTX-M-179, blaCTX-M-180, blaCTX-M-181, blaCTX-M-182, blaCTX-M-183, blaCTX-M-184, blaCTX-M-186, blaCTX-M-187, blaCTX-M-188, blaCTX-M-189, blaCTX-M-190, blaCTX-M-193, blaCTX-M-194, blaCTX-M-197, blaCTX-M-202, blaCTX-M-203, blaCTX-M-204, blaCTX-M-206, blaCTX-M-207, blaCTX-M-208, blaCTX-M-209, blaCTX-M-210, blaCTX-M-211, blaCTX-M-212, blaCTX-M-216, blaCTX-M-218, blaCTX-M-22, blaCTX-M-220, blaCTX-M-222, blaCTX-M-224, blaCTX-M-225, blaCTX-M-226, blaCTX-M-227, blaCTX-M-228, blaCTX-M-23, blaCTX-M-230, blaCTX-M-231, blaCTX-M-232, blaCTX-M-236, blaCTX-M-237, blaCTX-M-238, blaCTX-M-244, blaCTX-M-245, blaCTX-M-246, blaCTX-M-251, blaCTX-M-28, blaCTX-M-29, blaCTX-M-3, blaCTX-M-30, blaCTX-M-32, blaCTX-M-33, blaCTX-M-34, blaCTX-M-36, blaCTX-M-37, blaCTX-M-42, blaCTX-M-52, blaCTX-M-53, blaCTX-M-54, blaCTX-M-55, blaCTX-M-58, blaCTX-M-60, blaCTX-M-61, blaCTX-M-62, blaCTX-M-66, blaCTX-M-68, blaCTX-M-69, blaCTX-M-71, blaCTX-M-72, blaCTX-M-79, blaCTX-M-80, blaCTX-M-82, blaCTX-M-88, blaCTX-M-96	0.0	1.3	0.0	0.0	0.0
AMROTU_208	tet(Y)	0.0	1.3	0.0	0.0	0.0
AMROTU_982	catD, catP	0.0	1.2	0.0	0.0	0.0
AMROTU_990	catB3	0.0	1.2	0.0	0.0	0.0
AMROTU_976	catA2	0.0	1.2	0.0	0.0	0.0
AMROTU_219	tet(X3)	0.0	1.0	0.0	0.0	0.0
AMROTU_768	aadA7	0.0	0.9	0.0	0.0	0.0
AMROTU_683	blaOXA-1044, blaOXA-211, blaOXA-212, blaOXA-280, blaOXA-281, blaOXA-309, blaOXA-333, blaOXA-334, blaOXA-373, blaOXA-498, blaOXA-643, blaOXA-644, blaOXA-645, blaOXA-650, blaOXA-651, blaOXA-652, blaOXA-662	0.0	0.9	0.0	0.0	0.0
AMROTU_639	blaOXA-164, blaOXA-397, blaOXA-420, blaOXA-512, blaOXA-58, blaOXA-96, blaOXA-97	0.0	0.9	0.0	0.0	0.0
AMROTU_630	aadA11, aadA6, ant(3'')-Ia	0.0	0.9	0.0	0.0	0.0
AMROTU_464	blaAAK-1, blaOHIO-1, blaSHV-1, blaSHV-100, blaSHV-101, blaSHV-102, blaSHV-103, blaSHV-104, blaSHV-105, blaSHV-106, blaSHV-107, blaSHV-108, blaSHV-109, blaSHV-11, blaSHV-110, blaSHV-111, blaSHV-115, blaSHV-116, blaSHV-119, blaSHV-12, blaSHV-120, blaSHV-121, blaSHV-122b, blaSHV-128, blaSHV-129, blaSHV-13, blaSHV-132, blaSHV-133, blaSHV-134, blaSHV-135, blaSHV-137, blaSHV-14, blaSHV-141, blaSHV-142, blaSHV-143, blaSHV-144, blaSHV-145, blaSHV-146, blaSHV-147, blaSHV-148, blaSHV-149, blaSHV-15, blaSHV-150, blaSHV-151, blaSHV-152, blaSHV-153, blaSHV-154, blaSHV-155, blaSHV-156, blaSHV-157, blaSHV-158, blaSHV-159, blaSHV-16, blaSHV-160, blaSHV-161, blaSHV-162, blaSHV-163, blaSHV-164, blaSHV-165, blaSHV-168, blaSHV-171, blaSHV-172, blaSHV-173, blaSHV-178, blaSHV-179, blaSHV-18, blaSHV-180, blaSHV-182, blaSHV-183, blaSHV-185, blaSHV-186, blaSHV-187, blaSHV-188, blaSHV-189, blaSHV-190, blaSHV-191, blaSHV-193, blaSHV-194, blaSHV-195, blaSHV-196, blaSHV-197, blaSHV-198, blaSHV-199, blaSHV-1b-b, blaSHV-2, blaSHV-200, blaSHV-201, blaSHV-202, blaSHV-203, blaSHV-204, blaSHV-205, blaSHV-206, blaSHV-207, blaSHV-208, blaSHV-209, blaSHV-210, blaSHV-211, blaSHV-212, blaSHV-213, blaSHV-214, blaSHV-215, blaSHV-216, blaSHV-217, blaSHV-218, blaSHV-219, blaSHV-220, blaSHV-221, blaSHV-222, blaSHV-223, blaSHV-224, blaSHV-225, blaSHV-226, blaSHV-227, blaSHV-228, blaSHV-229, blaSHV-24, blaSHV-25, blaSHV-26, blaSHV-27, blaSHV-28,	0.0	0.9	0.0	0.0	0.0

	blaSHV-29, blaSHV-2A, blaSHV-3, blaSHV-30, blaSHV-31, blaSHV-32, blaSHV-33, blaSHV-34, blaSHV-35, blaSHV-36, blaSHV-37, blaSHV-38, blaSHV-4, blaSHV-40, blaSHV-41, blaSHV-42, blaSHV-43, blaSHV-44, blaSHV-45, blaSHV-46, blaSHV-48, blaSHV-49, blaSHV-5, blaSHV-50, blaSHV-51, blaSHV-52, blaSHV-55, blaSHV-56, blaSHV-57, blaSHV-59, blaSHV-60, blaSHV-61, blaSHV-62, blaSHV-63, blaSHV-64, blaSHV-65, blaSHV-66, blaSHV-67, blaSHV-69, blaSHV-7, blaSHV-70, blaSHV-71, blaSHV-72, blaSHV-73, blaSHV-74, blaSHV-75, blaSHV-76, blaSHV-77, blaSHV-78, blaSHV-79, blaSHV-8, blaSHV-80, blaSHV-81, blaSHV-82, blaSHV-85, blaSHV-86, blaSHV-89, blaSHV-9, blaSHV-92, blaSHV-93, blaSHV-94, blaSHV-95, blaSHV-96, blaSHV-97, blaSHV-98, blaSHV-99					
AMROTU_571	blaCARB-1, blaCARB-11, blaCARB-12, blaCARB-2, blaCARB-3, blaCARB-51, blaCARB-53, blaCARB-55, blaCARB-58, blaCARB-6	0.0	0.9	0.0	0.0	0.0
AMROTU_416	mph(A)	0.0	0.8	0.0	0.0	0.0
AMROTU_361	vanH-B	0.0	0.8	0.0	0.0	0.0
AMROTU_254	blaACT-106, blaACT-14, blaACT-15, blaACT-16, blaACT-17, blaACT-18, blaACT-19, blaACT-20, blaACT-21, blaACT-23, blaACT-24, blaACT-25, blaACT-27, blaACT-30, blaACT-31, blaACT-32, blaACT-33, blaACT-35, blaACT-36, blaACT-37, blaACT-39, blaACT-40, blaACT-41, blaACT-42, blaACT-43, blaACT-44, blaACT-45, blaACT-46, blaACT-47, blaACT-5, blaACT-55, blaACT-56, blaACT-59, blaACT-60, blaACT-61, blaACT-65, blaACT-66, blaACT-67, blaACT-69, blaACT-7, blaACT-70, blaACT-72, blaACT-73, blaACT-74, blaACT-75, blaACT-79, blaACT-83, blaACT-84, blaACT-85, blaACT-86, blaACT-89, blaACT-90	0.0	0.6	0.0	0.0	0.0
AMROTU_259	blaCFE-1, blaCMY-100, blaCMY-101, blaCMY-137, blaCMY-179, blaCMY-70, blaCMY-74, blaCMY-82, blaCMY-83, blaCMY-93	0.0	0.6	0.0	0.0	0.0
AMROTU_211	blaMCA	0.0	0.6	0.0	0.0	0.0
AMROTU_207	oqxA, oqxA10, oqxA11, oqxA2, oqxA3, oqxA4, oqxA5, oqxA6, oqxA7, oqxA8, oqxA9	0.0	0.6	0.0	0.0	0.0
AMROTU_158	cfr(C)	0.0	0.6	0.0	0.0	0.0
AMROTU_174	mef(A)	0.0	0.6	0.0	0.0	0.0
AMROTU_19	vanHBX	0.0	0.6	0.0	0.0	0.0
AMROTU_955	qnrS2, qnrS6	0.0	0.6	0.0	0.0	0.0
AMROTU_109	estX/sat2	0.0	0.5	0.0	0.0	0.0
AMROTU_758	blaOXA-118, blaOXA-119, blaOXA-205, blaOXA-46, blaOXA-779, blaOXA-835	0.0	0.5	0.0	0.0	0.0
AMROTU_84	mcr-9	0.0	0.5	0.0	0.0	0.0
AMROTU_75	mcr-1.1, mcr-1.10, mcr-1.11, mcr-1.12, mcr-1.13, mcr-1.14, mcr-1.15, mcr-1.16, mcr-1.17, mcr-1.18, mcr-1.19, mcr-1.2, mcr-1.20, mcr-1.21, mcr-1.22, mcr-1.23, mcr-1.24, mcr-1.25, mcr-1.26, mcr-1.27, mcr-1.28, mcr-1.29, mcr-1.3, mcr-1.30, mcr-1.31, mcr-1.32, mcr-1.33, mcr-1.34, mcr-1.4, mcr-1.5, mcr-1.6, mcr-1.7, mcr-1.8, mcr-1.9	0.0	0.5	0.0	0.0	0.0
AMROTU_515	mph(G)	0.0	0.4	0.0	0.0	0.0

AMROTU_403	blaPER-1, blaPER-10, blaPER-11, blaPER-12, blaPER-13, blaPER-15, blaPER-16, blaPER-3, blaPER-4, blaPER-5, blaPER-7, blaPER-8, blaPER-9	0.0	0.4	0.0	0.0	0.0
AMROTU_238	blaBIL-1, blaCMY-102, blaCMY-103, blaCMY-104, blaCMY-105, blaCMY-106, blaCMY-107, blaCMY-108, blaCMY-109, blaCMY-110, blaCMY-111, blaCMY-112, blaCMY-113, blaCMY-114, blaCMY-115, blaCMY-116, blaCMY-117, blaCMY-118, blaCMY-119, blaCMY-12, blaCMY-121, blaCMY-122, blaCMY-124, blaCMY-125, blaCMY-127, blaCMY-128, blaCMY-129, blaCMY-13, blaCMY-130, blaCMY-131, blaCMY-132, blaCMY-133, blaCMY-134, blaCMY-135, blaCMY-136, blaCMY-138, blaCMY-139, blaCMY-14, blaCMY-140, blaCMY-141, blaCMY-142, blaCMY-143, blaCMY-144, blaCMY-145, blaCMY-146, blaCMY-147, blaCMY-148, blaCMY-149, blaCMY-15, blaCMY-150, blaCMY-151, blaCMY-152, blaCMY-153, blaCMY-154, blaCMY-155, blaCMY-156, blaCMY-158, blaCMY-16, blaCMY-160, blaCMY-161, blaCMY-162, blaCMY-163, blaCMY-164, blaCMY-165, blaCMY-166, blaCMY-167, blaCMY-168, blaCMY-169, blaCMY-17, blaCMY-170, blaCMY-171, blaCMY-172, blaCMY-173, blaCMY-174, blaCMY-175, blaCMY-176, blaCMY-177, blaCMY-18, blaCMY-2, blaCMY-20, blaCMY-21, blaCMY-22, blaCMY-23, blaCMY-24, blaCMY-25, blaCMY-26, blaCMY-27, blaCMY-28, blaCMY-29, blaCMY-2b, blaCMY-30, blaCMY-31, blaCMY-32, blaCMY-33, blaCMY-34, blaCMY-35, blaCMY-36, blaCMY-37, blaCMY-38, blaCMY-39, blaCMY-4, blaCMY-40, blaCMY-41, blaCMY-42, blaCMY-43, blaCMY-44, blaCMY-45, blaCMY-46, blaCMY-47, blaCMY-48, blaCMY-49, blaCMY-5, blaCMY-50, blaCMY-51, blaCMY-53, blaCMY-54, blaCMY-55, blaCMY-56, blaCMY-57, blaCMY-58, blaCMY-59, blaCMY-6, blaCMY-60, blaCMY-61, blaCMY-62, blaCMY-63, blaCMY-64, blaCMY-65, blaCMY-66, blaCMY-67, blaCMY-68, blaCMY-69, blaCMY-7, blaCMY-71, blaCMY-72, blaCMY-73, blaCMY-75, blaCMY-76, blaCMY-77, blaCMY-78, blaCMY-79, blaCMY-80, blaCMY-81, blaCMY-84, blaCMY-85, blaCMY-86, blaCMY-87, blaCMY-89, blaCMY-90, blaCMY-94, blaCMY-95, blaCMY-96, blaCMY-97, blaCMY-99, blaLAT-1, blaLAT-3	0.0	0.3	0.0	0.0	0.0
AMROTU_190	cml	0.0	0.3	0.0	0.0	0.0
AMROTU_152	cmlA, cmlA1, cmlA10, cmlA4, cmlA5, cmlA6, cmlA8	0.0	0.3	0.0	0.0	0.0
AMROTU_112	lsa(E)	0.0	0.2	0.0	0.0	0.0
AMROTU_6	tmexD1, tmexD2, tmexD3	0.0	0.2	0.0	0.0	0.0
	**Total RPKM**	**548.7**	**684.0**	**0**	**0**	**0**

RPKM: Reads Per Kilobase of gene per Million.
