# Supplementary material for: Inactivation of Bacteria and Residual Antimicrobials in Hospital Wastewater by Ozone Treatment
Source: Antibiotics (Basel). 2022 Jun 27;11(7):862. doi: 10.3390/antibiotics11070862 (PMC9311624; doi:10.3390/antibiotics11070862)
Supplement: Supplementary file 1 [file antibiotics-11-00862-s001.zip › Supplementary-Table-S4.pdf]

antibiotics

MDPI

Supplementary Materials:

Table S4. Whole genome sequence of bacteria isolated using CHROMagar ESBL after ozone treatment.

Ozone treatment (min)	Strain Name	CHROMagar ESBL (colony color)	Total Base	Predicted Organism by Kraken	Putative Serotype	MLST	Antimicrobial Resistance Gene	DDBJ Accession number
0	**STN211208-OZ-0m-01**	pink	5,173,467	*Escherichia coli*	O16:H5	ST: 131	aac(3)-IId, aadA5, aph(3'')-Ib, aph(6)-Id, blaCTX-M-15, blaEC-5, blaTEM-1, dfrA17, mdf(A), mph(A), qacEdelta1, sitABCD, sul1, sul2, tet(A)	DRR376744
0	**STN211208-OZ-0m-02**	pink	5,095,401	*Escherichia coli*	O25:H4	ST: 131	blaCTX-M-27, blaEC-5, mdf(A), sitABCD	DRR376745
0	**STN211208-OZ-0m-03**	pink	5,095,705	*Escherichia coli*	O25:H4	ST: 131	blaCTX-M-27, blaEC-5, mdf(A), sitABCD	DRR376746
0	**STN211208-OZ-0m-04**	pink	5,147,020	*Aeromonas caviae*	-	-	aadA2, aph(3'')-Ib, aph(6)-Id, blaCTX-M-3, blaMOX-2, blaOXA-1037, qacEdelta1, sul1	DRR376747
0	**STN211208-OZ-0m-05**	pink	5,215,571	*Escherichia coli*	O25:H4	ST: 131	blaCTX-M-14, blaEC-5, mdf(A), sitABCD	DRR376748
0	**STN211208-OZ-0m-06**	pink	5,097,760	*Escherichia coli*	O25:H4	ST: 131	blaCTX-M-27, blaEC-5, mdf(A), sitABCD	DRR376749
0	**STN211208-OZ-0m-07**	Dark blue	6,108,121	*Raoultella ornithinolytica*	-	-	aadA2, aph(3'')-Ib, aph(6)-Id, blaCTX-M-62, blaGES-24, blaORN-3, fosA, mph(A)	DRR376750
0	**STN211208-OZ-0m-08**	Dark blue	4,952,835	*Aeromonas hydrophila*	-	-	aac(6')-31, aph(3'')-Ib, aph(3')-VI, blaGES-11, blaOXA-950, catB8, cepS, cphA1, sul1, tet(C)	DRR376751
0	**STN211208-OZ-0m-09**	Dark blue	4,907,551	*Aeromonas hydrophila*	-	-	aac(6')-31, aac(6')-II, aph(3')-VI, blaGES-24, blaOXA-950, cepS, cphA2, tet(C)	DRR376752
0	**STN211208-OZ-0m-10**	Dark blue	5,375,982	*Klebsiella pneumoniae*	-	ST: 101	aph(3'')-Ib, aph(6)-Id, blaCTX-M-15, blaSHV-212, blaTEM-1, dfrA14, fosA, oqxA10, oqxB17, qnrB1, sul2	DRR376753
0	**STN211208-OZ-0m-11**	Dark blue	5,028,805	*Citrobacter freundii*	-	-	aac(6')-31, aac(6')-IIc, blaCMY-65, blaGES-5, blaIMP-1, fosE, qacEdelta1, qnrB38	DRR376754
0	**STN211208-OZ-0m-12**	White	4,960,244	*Aeromonas hydrophila*	-	-	aac(6')-31, aph(3'')-Ib, aph(3')-VI, blaGES-3, blaOXA-950, catB8, cepS, cphA1, qacEdelta1, sul1, tet(C)	DRR376755
0	**STN211208-OZ-0m-13**	Dark blue	6,005,525	*Raoultella ornithinolytica*	-	-	aadA2, aph(3'')-Ib, aph(6)-Id, blaCTX-M-62, blaGES-24, blaORN-3, fosA	DRR376756
0	**STN211208-OZ-0m-14**	Dark blue	6,103,013	*Raoultella ornithinolytica*	-	-	aadA2, aph(3'')-Ib, aph(6)-Id, blaCTX-M-62, blaGES-24, blaORN-3, fosA, mph(A)	DRR376757
0	**STN211208-OZ-0m-15**	Dark blue	6,315,435	*Raoultella ornithinolytica*	-	-	aadA2, blaCTX-M-62, blaORN-3, fosA, qacEdelta1, sul1	DRR376758
0	**STN211208-OZ-0m-16**	White	3,990,885	*Acinetobacter sp.*	-	-	blaMCA, blaOXA-58, mph(E), msr(E), tet(39)	DRR376759
0	**STN211208-OZ-0m-17**	White	6,593,438	*Pseudomonas putida*	-	-	aac(6')-II, qacEdelta1, sul1	DRR376760
0	**STN211208-OZ-0m-18**	White	6,379,242	*Pseudomonas putida*	-	-	-	DRR376761
10	**STN211208-OZ-10m-01**	pink	5,095,015	*Escherichia coli*	O25:H4	ST: 131	blaCTX-M-27, blaEC-5, mdf(A), sitABCD	DRR376762
10	**STN211208-OZ-10m-02**	pink	5,120,949	*Aeromonas caviae*	-	-	aadA2, aph(3'')-Ib, aph(6)-Id, blaCTX-M-3, blaMOX-2, blaOXA-1037, mph(A), qacEdelta1, sul1	DRR376763
10	**STN211208-OZ-10m-03**	pink	5,267,883	*Escherichia coli*	O1:H6	ST: 648	blaCTX-M-14, blaEC-19, mdf(A)	DRR376764
10	**STN211208-OZ-10m-04**	pink	5,093,806	*Escherichia coli*	O25:H4	ST: 131	blaCTX-M-27, blaEC-5, mdf(A), sitABCD	DRR376765
10	**STN211208-OZ-10m-05**	Dark blue	5,575,818	*Citrobacter freundii*	-	-	aadA2, aph(3'')-Ib, aph(6)-Id, blaCMY-70, blaGES-24, mph(A), qacEdelta1, sul1	DRR376766
10	**STN211208-OZ-10m-06**	Dark blue	5,931,276	*Raoultella ornithinolytica*	-	-	aadA2, aph(3'')-Ib, aph(6)-Id, blaCTX-M-62, blaGES-24, blaORN-3, fosA	DRR376767
10	**STN211208-OZ-10m-07**	Dark blue	4,948,480	*Aeromonas caviae*	-	-	ARR-3, aac(6')-31, ant(3'')-Ij/aac(6')-Ib, blaGES-3, blaMOX-2, blaOXA-1037, blaPER-3, catB3, dfrA27, mph(A), qnrVC6	DRR376768
10	**STN211208-OZ-10m-08**	White	4,953,590	*Aeromonas hydrophila*	-	-	aac(6')-31, aph(3'')-Ib, aph(3')-VI, blaGES-1, blaOXA-950, catB8, cepS, cphA1, qacEdelta1, sul1, tet(C)	DRR376769
10	**STN211208-OZ-10m-09**	Dark blue	6,422,989	*Raoultella ornithinolytica*	-	-	aadA2, blaCTX-M-62, blaGES-24, blaORN-3, fosA, qacEdelta1, sul1	DRR376770
10	**STN211208-OZ-10m-10**	Dark blue	5,988,785	*Raoultella ornithinolytica*	-	-	aadA2, aph(3'')-Ib, aph(6)-Id, blaCTX-M-62, blaGES-24, blaORN-3, fosA	DRR376771
10	**STN211208-OZ-10m-11**	Dark blue	5,614,682	*Citrobacter freundii*	-	-	aac(6')-IIc, aadA5, aph(3')-Ib, blaCMY-70, blaIMP-6, blaSHV-12, qnrA1, sul1, tet(A)	DRR376772
10	**STN211208-OZ-10m-12**	Dark blue	5,971,655	*Raoultella ornithinolytica*	-	-	aadA2, aph(3'')-Ib, aph(6)-Id, blaCTX-M-62, blaGES-24, blaORN-3, fosA	DRR376773
10	**STN211208-OZ-10m-13**	Dark blue	6,027,156	*Raoultella ornithinolytica*	-	-	blaCTX-M-62, blaGES-24, blaORN-4, fosA, qacEdelta1, sul1	DRR376774
10	**STN211208-OZ-10m-14**	Dark blue	5,996,388	*Raoultella ornithinolytica*	-	-	aadA2, aph(3'')-Ib, aph(6)-Id, blaCTX-M-62, blaGES-24, blaORN-3, fosA	DRR376775
10	**STN211208-OZ-10m-15**	Dark blue	5,986,254	*Raoultella ornithinolytica*	-	-	aadA2, aph(3'')-Ib, aph(6)-Id, blaCTX-M-62, blaGES-24, blaORN-3, fosA	DRR376776
10	**STN211208-OZ-10m-16**	White	5,891,451	*Pseudomonas nitroreducens*	-	-	-	DRR376777
10	**STN211208-OZ-10m-17**	White	6,889,360	*Pseudomonas nitroreducens*	-	-	-	DRR376778
10	**STN211208-OZ-10m-18**	White	6,174,373	*Pseudomonas putida*	-	-	-	DRR376779
20	**STN211208-OZ-20m-01**	Dark blue	6,152,737	*Raoultella ornithinolytica*	-	-	blaCTX-M-62, blaGES-24, blaORN-4, fosA, qacEdelta1, sul1	DRR376780
20	**STN211208-OZ-20m-02**	Dark blue	6,105,280	*Raoultella ornithinolytica*	-	-	aadA2, aph(3'')-Ib, aph(6)-Id, blaCTX-M-62, blaGES-24, blaORN-3, fosA, mph(A)	DRR376781
20	**STN211208-OZ-20m-03**	Dark blue	4,967,623	*Aeromonas hydrophila*	-	-	aac(6')-31, aph(3'')-Ib, aph(3')-VI, blaGES-4, blaOXA-950, catB8, cepS, cphA1, sul1, tet(C)	DRR376782
20	**STN211208-OZ-20m-04**	Dark blue	5,589,090	*Citrobacter freundii*	-	-	aac(6')-IIc, aadA5, aph(3')-Ib, blaCMY-70, blaIMP-6, blaSHV-12, qnrA1, sul1, tet(A)	DRR376783
20	**STN211208-OZ-20m-05**	Dark blue	6,100,242	*Raoultella ornithinolytica*	-	-	aadA2, aph(3'')-Ib, aph(6)-Id, blaCTX-M-62, blaGES-24, blaORN-3, fosA, mph(A)	DRR376784
20	**STN211208-OZ-20m-06**	Dark blue	6,184,381	*Raoultella ornithinolytica*	-	-	blaCTX-M-62, blaGES-24, blaORN-4, fosA, qacEdelta1, sul1	DRR376785
20	**STN211208-OZ-20m-07**	Dark blue	10,083,410	*Raoultella ornithinolytica*	-	-	aadA2, aph(3'')-Ib, aph(6)-Id, blaCTX-M-62, blaGES-24, blaORN-3, fosA, mph(A)	DRR376786
20	**STN211208-OZ-20m-08**	Dark blue	5,523,946	*Citrobacter freundii*	-	-	aadA5, blaCMY-70, blaSHV-12, qnrA1, sul1	DRR376787
20	**STN211208-OZ-20m-09**	White	6,173,264	*Pseudomonas putida*	-	-	-	DRR376788
20	**STN211208-OZ-20m-10**	White	6,589,339	*Pseudomonas putida*	-	-	aac(6')-II, qacEdelta1, sul1	DRR376789
20	**STN211208-OZ-20m-11**	White	6,172,290	*Pseudomonas putida*	-	-	-	DRR376790
20	**STN211208-OZ-20m-12**	White	6,173,140	*Pseudomonas putida*	-	-	-	DRR376791
40	**STN211208-OZ-40m-01**	Dark blue	6,398,913	*Raoultella ornithinolytica*	-	-	aadA2, blaCTX-M-62, blaORN-3, fosA, qacEdelta1, sul1	DRR376792
40	**STN211208-OZ-40m-02**	Dark blue	6,098,754	*Raoultella ornithinolytica*	-	-	aadA2, aph(3'')-Ib, aph(6)-Id, blaCTX-M-62, blaGES-24, blaORN-3, fosA, mph(A)	DRR376793
40	**STN211208-OZ-40m-03**	Dark blue	6,094,173	*Raoultella ornithinolytica*	-	-	blaCTX-M-62, blaGES-24, blaORN-4, fosA, qacEdelta1, sul1	DRR376794
40	**STN211208-OZ-40m-04**	Dark blue	5,986,315	*Raoultella ornithinolytica*	-	-	aadA2, aph(3'')-Ib, aph(6)-Id, blaCTX-M-62, blaGES-24, blaORN-3, fosA	DRR376795
40	**STN211208-OZ-40m-05**	White	6,173,926	*Pseudomonas putida*	-	-	-	DRR376796
40	**STN211208-OZ-40m-06**	White	6,305,021	*Pseudomonas putida*	-	-	-	DRR376797
80	**STN211208-OZ-80m-01**	Dark blue	6,103,859	*Raoultella ornithinolytica*	-	-	aadA2, aph(3'')-Ib, aph(6)-Id, blaCTX-M-62, blaGES-24, blaORN-3, fosA, mph(A)	DRR376798
80	**STN211208-OZ-80m-02**	White	6,172,375	*Pseudomonas putida*	-	-	-	DRR376799
80	**STN211208-OZ-80m-03**	White	6,172,741	*Pseudomonas putida*	-	-	-	DRR376800
80	**STN211208-OZ-80m-04**	White	6,593,522	*Pseudomonas putida*	-	-	aac(6')-II, qacEdelta1, sul1	DRR376801
80	**STN211208-OZ-80m-05**	White	6,608,760	*Pseudomonas putida*	-	-	aac(6')-II, qacEdelta1, sul1	DRR376802
80	**STN211208-OZ-80m-06**	White	6,218,308	*Pseudomonas putida*	-	-	-	DRR376803
